# Correlates of Early-Stage Frailty—Sleep, Fitness, Oxidative Stress, and BMI

**DOI:** 10.3389/fmed.2020.594710

**Published:** 2021-01-13

**Authors:** Yael Netz, Sigal Ben-Zaken, Aviva Zeev, Ayelet Dunsky

**Affiliations:** The Academic College at Wingate, Wingate Institute, Netanya, Israel

**Keywords:** frailty attenuation, pre-frailty, cardiorespiratory fitness, physical activity, sleep quality, hydrogen peroxide, deficit accumulation

## Abstract

Frailty reflects a decreased reserve in multiple systems resulting from cumulative decline. Frailty markers should be identified as early as possible to attenuate the loss of reserve. The aim of this study was to identify potentially modifiable correlates of frailty in relatively healthy older adults. Volunteers (*n* = 122) were recruited from local councils and, based on gender and age, were divided into one group of men aged 77.0 (±5.3), and two groups of women, aged 68.8 (±3.6) and aged 78.4 (±3.4). Frailty was assessed by a Frailty Index. The examined correlates were: physical activity, physical fitness (predicted peak VO_2_), sleep quality, oxidative stress (hydrogen peroxide—H_2_O_2_) and depression. Both groups of women had poor scores on physical fitness compared to women's norms. In order to examine the contribution of each of the potential correlates to explaining the variance of frailty, stepwise regressions were performed for each group separately. Based on the results, none of the suggested correlates significantly explained the variability of frailty in the men. In the younger-aged women, predicted peak VO_2_ and sleep quality explained 22.4% of the variability of frailty. In the older women, Body Mass Index (BMI), oxidative stress and sleep quality explained 34.9% of the variance. It is possible that increased aerobic fitness and interventions for improving sleep quality in older, seemingly healthy women will slow down the frailty process. Further research is needed to assess potential correlates of frailty, and to initiate suitable interventions to mitigate the signs of frailty at an early stage.

## Introduction

Frailty is a state of increased vulnerability to adverse health outcomes, such as falls (1), hospitalization (2), and death (3), which develops as a consequence of age-related decline (4). However, trajectories of health in aging can vary significantly depending on the individual, reflecting the heterogeneity in health among people of the same age (5), and emphasizing the need to quantify these losses (6).

In a comprehensive review of definitions of frailty based on the literature, along with expert opinions of official bodies, there was no consensus on a definition of frailty, but there was agreement that disability is a consequence rather than the cause of frailty; thus, frailty is considered as a pre-disability stage (7). Although it appears that frailty is to a large extent associated with disability and comorbidity, it was argued that these are conceptually distinct (8), one of the differences being that frailty is recognized as a potentially modifiable state (6). More specifically, research has demonstrated that the nature of frailty is dynamic and can be reversed at certain levels, thus early intervention should be considered (9–11).

Consequently, it is important to identify as early as possible those frailty markers which are potentially modifiable, in order to either attenuate the loss of reserve or even reverse it. Therefore, while quite a few studies focus on frailty status as related to major hazards [e.g., (1–3)], our approach in this study is to examine its relation to potentially modifiable variables at an early stage of frailty, when the chances of reducing the vulnerability to adverse health outcomes are higher.

In the current study, we evaluated correlates of frailty in healthy and active people 65+. Based on a literature review, we chose to assess the following potential correlates: sleep quality, structured physical activity, physical fitness (predicted peak VO_2_), hydrogen peroxide (H_2_O_2_)—an operative in redox sensing and redox signaling, BMI (Body Mass Index) and depression.

Two systematic reviews have recently been published examining the relationship between frailty and sleep. One reported a consistent relationship between frailty and sleep disturbances, although the review was based on limited research (12). The other focused on sleep duration, suggesting that while shorter sleep duration (under 6 h) was associated with risk of frailty, prolonged sleep time (more than 8 h) was significantly related to enhanced risk of pre-frailty (13).

Epidemiological studies on the impact of physical activity on frailty levels suggested an association between physical activity and a lower risk of either developing or mitigating the severity of frailty (14). While physical activity and physical fitness are clearly related, they are not identical. Cardiorespiratory fitness is the gold standard measure of assessing physical fitness. It has been shown that low cardiorespiratory fitness, defined as either maximal (VO_2_max) or submaximal oxygen uptake, created relative risks to mortality comparable to those for smoking, hypercholesterolemia, hypertension, family history of coronary heart disease, BMI and an elevated serum glucose level (15). On the other hand, studies exploring the relationship between oxygen uptake and frailty are scarce. A recent long-term observational study reported that VO_2_max was useful in mortality prediction, based on frailty stratification (16).

One aspect of age deterioration is expressed by the accumulation of cellular damage. More specifically, the balance between the cellular antioxidant defense system and toxic effects due to oxidative stress may deteriorate (17), resulting in oxidative stress (18). Oxidative stress produces a variety of highly reactive free radicals that damage cells, initiate signal transduction pathways and alter gene expression. Cells are capable of countering the effects of oxidative stress by virtue of a complex redox buffering system. An accumulating body of evidence has suggested that oxidative stress and inflammatory changes might play a role in the development of frailty (19). A systematic review on oxidative stress and frailty showed clear evidence of higher levels of peripheral oxidative stress biomarkers and lower anti-oxidant parameters among frail as compared to non-frail older adults (20). However, the effects of both oxidative stress and anti-oxidant parameters on the initiation and progression of age-related diseases remain unclear. Hydrogen peroxide (H_2_O_2_) has emerged as the major redox metabolite operative in redox sensing, signaling and redox regulation (21). H_2_O_2_ serves fundamental regulatory functions in metabolism beyond its role as a damage signal (22). Since H_2_O_2_ can be detected in freshly-voided human urine, it has been proposed as a non-invasive “biomarker” of oxidative stress (23).

Another variable that has been mentioned in the literature in relation to frailty is BMI (24). While the association between weight loss and low BMI and frailty has been acknowledged and therefore is included in the leading frailty instruments (25, 26), some studies also found an association between obesity and frailty (24). A more recent review indicated that not only underweight but also obesity is associated with frailty (27).

Not surprisingly, depression has also been investigated in relation to frailty. A systematic review on frailty and depression indicated that the incidence of depressive symptomatology or frailty, or their co-occurrence, was higher than 10% in older adults aged 55+ and that a high percentage of older adults who are frail also have depressive symptomatology (28).

Considering the importance of identifying frailty markers as early as possible, the purpose of this study was to determine to what extent the above-mentioned variables explain the variability of frailty in older adults who are considered to be healthy, and to identify the correlates which are more dominant in explaining this variability.

## Methods

### Participants

This study was based on community-dwelling volunteers; the recruitment and measurement process lasted ~6 months. We recruited 123 healthy and active community-dwelling older adults (age 65+) from local councils and sports clubs, as well as through ads on Facebook. All of the chosen participants had been physically active at least once a week for at least 3 months prior to the study, and were required to be able to perform a maximal exercise test. All participants had a score of >24 on the Mini-Mental State Examination—MMSE (29), excluding one person who had a score of 22. Ethical approval for the study was obtained from the Ethics Committee of the Hillel Yaffe Medical Center (Hadera, Israel), and a written consent was obtained from all participants. One person was unable to complete the maximal exercise test, leaving 122 (36 men) participants.

As the men were significantly older than the women, and women outnumbered men, the women were divided into two age groups, with the median age as the cutoff point. The cutoff point was age 75 (young <74, old ≥75), which actually fits the traditional cutoff point between “young-old” and “old” [e.g., (30)].

### Measurements

#### Frailty

The Frailty Index used in this study is based on a deficit accumulation approach, suggesting that the additive effect of even smaller health problems may yield poor overall health (26). Variables are considered as deficits and contribute to a frailty index if they are associated with health status, cover a range of systems and their prevalence generally increases with age. The list of deficits may vary in the various frailty indexes, but for any given frailty index the value of the index, using n variables, is defined for a given individual as the fraction x/n, where x is the number of deficits recorded for that person (6).

In the current study we used a Frailty Index (FI) suggested by Rockwood et al. (6). It comprises 48 items representing deficits in various areas of functioning: pathologies, mental status (MMSE), emotional status (depression, anxiety), communication (hearing, vision etc.), mobility, balance, daily activities, bladder and bowl functions, sleep, and social resources. Based on the equation, a person with 8 deficits, for example, has a score of 0.17. Although it is a continuous measure ranging from 0 to a theoretical maximum of 1.0 (6), there appears to be a limit to the number of health deficits people can tolerate, thus the range of the upper end of the scale lies between 0.4 (31), and 0.7 (32), and it is obviously higher among clinical populations and the very old people.

#### Physical Activity

The short form of the International Physical Activity Questionnaire—IPAQ-SF (33), examining 7-day recall of physical activity, was used to assess habitual physical activity. The IPAQ-SF inquires about the duration (in minutes) and frequency (days) of three types of activities: walking, moderate-intensity activities and vigorous-intensity activities, as well as about inactivity (time spent sitting). Each activity is weighted by its energy requirements, defined in Metabolic Equivalents (METs) based on the compendium of activities (34).

The scores are calculated as MET-min per week: MET level × minutes of activity/day × days per week. The formula for total MET Continuous score-minutes/week = Walking (3.3 METs*min*days) + Moderate Intensity (4 METs*min*days) + Vigorous Intensity (8 METs*min*days). According to the WHO guidelines 600 METs is the minimum required for health purposes, and is considered low-level exercise (<600 is considered insufficient exercise). A level of 600 to 3,000 METs is considered a moderate exercise level, and more than 3,000 a high level of exercise (35).

#### Physical Fitness

A graded, progressive, maximal exercise test was administered to assess fitness level (predicted peak VO_2_) and maximal heart rate (HRmax). Participants performed the test on a motorized treadmill (Woodway, Germany). For the duration of the test, the electrocardiogram (ECG), heart rate (HR), blood pressure and rating of perceived exertion of the participants were continuously monitored, using a 12-lead ECG, a sphygmomanometer and the Borg scale (36), respectively. The test commenced with 2–5 min of practice and adaptation. Based on the modified Balke protocol (37), an initial speed of 3.2 km/h with a gradient of zero was determined. The gradient was increased by 2.5% every 2 min until a negative symptom appeared (e.g., dizziness, breathlessness, changes in ECG), limited max was reached, or until the participant reached his/her limit of tolerance. Peak VO_2_ was estimated from the last stage of the graded maximal treadmill exercise challenge. The following equation was used to estimate peak VO_2_ for each subject: VO_2_ (ml*kg^−1^*min^−1^) = 0.1 (final speed) + 1.8 (final speed) (final fractional grade) + 3.5 ml* kg^−1^*min^−1^ (37).

#### Pittsburgh Sleep Quality Index (PSQI)

The PSQI (38) is a validated, widely-used measure of subjective sleep quality and sleep disturbances over a 1-month time period. It consists of seven sleep components (sleep quality, sleep latency, sleep duration, sleep efficiency, sleep disturbances, use of sleeping medication, daytime dysfunction) on a 0 (no difficulty) to 3 (severe difficulty) scale, resulting in a global score between 0 and 21. A score of >5 is indicative of poor self-reported sleep quality.

#### Geriatric Depression Scale (GDS)

We used the GDS-15 (39), which consists of 15 items. Each of the items has two possible answers (yes or no). A score of 0–2 on the GDS is considered to be “no depression” and 3–6 “partial depression.”

#### Hydrogen Peroxide (H_2_O_2_)

Mid-stream urine samples were collected into plain containers and were frozen until analysis. Urine samples were centrifuged in 4C for 8 min at 2,500 rpm. H_2_O_2_ was detected by fluorometric assay using Hydrogen Peroxide Assay Kit (abcam https://www.abcam.com/ : ab102500) according to the manufacturer's protocol. The assay is based on the reaction between horseradish peroxidase (HRP) with a probe and H_2_O_2_ to produce a product with color (λmax = 570 nm) and red-fluorescence (Ex/Em = 535/587 nm). The detection limit is 2 pmol per assay (or 40 nM concentration) of H_2_O_2_ in the sensitive fluorometric assay.

### Procedure

Participants visited the laboratory individually. They first provided their written informed consent. Their demographic and clinical data, as well as the MMSE, GDS, IPAQ-SF, and PSQI data, were collected by the research team. They were then asked to provide a urine sample for the H_2_O_2_ assessment. A physician interviewed them for the FI, and finally they performed the predicted peak VO_2_ test in the presence of a supervising physician. The BMI was calculated based on weight and height values measured prior to the fitness test.

## Results

Table 1 presents the means (±SDs) of the background and the study variables for the three study groups: men, young-old women and old women. Except for the expected gender differences on height and weight, the men reported significantly more physical activity (IPAQ-SF) than the young-old women, and their predicted peak VO_2_–relative to age and gender norms (40)—was significantly higher than that of both age groups of women. No significant group differences were detected on frailty, although the men and the old women had a higher rate of frailty (0.10) as compared to the young-old women (0.08).

**Table 1:** Means (+SDs) of the background and the study variables for the three study groups.

	**Men**	**Young-old women**	**Old women**
	**A**	**B**	**C**
N	36	44	42
Age (years)	77.0 (5.3) ^>B^^*^	68.8 (3.6)	78.4 (3.4)^>B^^*^
Weight (kg)	77.9 (9.7) ^>B, >C^^*^	69.9 (11.1)	66.9 (10.3)
Height (m)	1.7 (0.1) ^>B, >C^^*^	1.6 (0.1)	1.6 (0.1)
BMI (kg/m^2^)	26.4 (3.5)	27.9 (4.0)	26.9 (3.9)
MMSE	28.7 (1.8)	28.9 (1.3)	29.4 (1.2)
GDS	1.7 (2.0)	2.8 (2.9)	1.7 (1.8)
Education (years)	14.0 (3.2)	12.9 (4.0)	12.8 (2.4)
IPAQ (METs)	2,246.5 (1430.1) ^>B^^*^	1,447.3 (855.5)	1,782.5 (779.3)
Predicted peak VO_2_ (ml^*^kg^−1^^*^min^−1^)	29.7 (6.9) ^>B>C^^*^^1^	23.8 (7.4)	21.3 (7.0)
H_2_O_2_	11.8 (4.0)	11.3 (4.0)	11.7 (3.7)
PSQI	4.5 (2.6)	5.6 (3.3)	5.2 (3.6)
FI	0.10 (0.06)	0.08 (0.05)	0.10 (0.06)

* *P < 0.05*.

*BMI, Body Mass Index; MMSE, Mini Mental State Examination; GDS, Geriatric Depression Scale; IPAQ, International Physical Activity Questionnaire; METs, Metabolic Equivalents; H_2_O_2_, Hydrogen peroxide; PSQI, The Pittsburgh Sleep Quality Index; FI, Frailty Index*.

1 *Based on predicted VO_2_ max norms of Cooper Clinic data (40), men reached (approx). the 55th percentile, young women the 15th, and old women the 12th percentile*.

Table 2 reports the correlations between frailty (FI) and the other study variables: physical activity—METs (IPAQ-SF), physical fitness (predicted peak VO_2_), BMI, oxidative stress (H_2_O_2_), sleep disturbances (PSQI), and depression (GDS). Frailty significantly correlated with physical fitness and sleep disturbances in the young-old women (*p* < 0.05) and with BMI (*p* < 0.05) in the old women, and the correlation with oxidative stress was marginally significant (*p* = 0.056) in the old women. The correlation with sleep disturbances in the old women was relatively high, although it did not reach significance. In the men, none of the correlations reached significance. Regarding interrelations—depression was significantly correlated with sleep quality in both the men (*R* = 0.41, *p* = 0.01) and the young-old women (*R* = 0.32, *p* < 0.05), and was marginally significant with oxidative stress in the old women (*R* = 0.29; *p* = 0.06); physical activity was correlated with physical fitness in the young-old women (*R* = 0.34; *p* < 0.03).

**Table 2:** Correlations between frailty and physical fitness (Predicted peak VO_2_), physical activity (METs), BMI, oxidative stress (H_2_O_2_), sleep disturbances (PSQI), and depression (GDS).

	**Frailty (FI)**		
	**Men**	**Young-old women**	**Old women**
**Variable**			
Predicted peak VO_2_ (ml^*^kg^−1^^*^min^−1^)	−0.044	−0.308^*^	−0.047
IPAQ (METs)	−0.201	−0.247	−0.038
BMI	−0.058	−0.031	0.362^*^
H_2_O_2_	0.084	0.107	0.297^°^
PSQI	0.208	0.300^*^	0.279
GDS	0.200	0.072	0.138

*IPAQ, International Physical Activity Questionnaire; METs, Metabolic Equivalents; BMI, Body Mass Index; H_2_O_2_, Hydrogen peroxide; PSQI, The Pittsburgh Sleep Quality Index; GDS, Geriatric Depression Scale*.

* *P ≤ 0.05*.

° *P = 0.056*.

Regression analyses including all variables collectively (IPAQ-SF, predicted peak VO_2_, PSQI, H_2_O_2_, BMI, GDS), performed separately for the three groups, indicated a shared variability with frailty of 9.3% (*R*^2^ = 0.093) in the men, 27.4% in the young-old women and 36.1% in the old women. In stepwise regressions no variables entered the regression for the men. Table 3 presents the results for the women. In the young-old women, predicted peak VO_2_ (fitness) entered first the regression followed by PSQI (sleep quality), both explaining 22.4% of the variability of frailty. In the old women BMI followed by oxidative stress (H_2_O_2_), and PSQI explained 34.9% of the variance.

**Table 3:** Summary of stepwise regressions for frailty, for women.

			**95.0% Confidence Interval**	
**Variables entered**	***R*^**2**^**	**B**	**Lower bound**	**Upper bound**
**Young Women**				
Predicted peak VO_2_ (Fitness)	0.095	−0.003	−0.005	−0.001
PSQI (Sleep Quality)	0.224	0.006	0.001	0.010
**Old Women**				
BMI	0.131	0.006	0.002	0.010
H_2_O_2_ (Oxidative Stress)	0.239	0.006	0.001	0.010
PSQI (Sleep Quality)	0.349	0.005	0.001	0.010

*BMI, Body Mass Index; H_2_O_2_, Hydrogen peroxide; PSQI, The Pittsburgh Sleep Quality Index*.

## Discussion

Given that frailty markers should be detected as early as possible (9–11), our study is unique in that it focuses on correlates of frailty in old people with a low level of frailty [0.10 for age 75+ as compared to ~0.16 reported in the literature for that age; see (31)].

Based on our results, physical fitness (predicted peak VO_2_) and sleep quality were significant predictors of frailty in young-old women. The importance of physical fitness in the prevention of chronic diseases as well as premature death is well-documented [e.g., (15, 16), respectively]. In this study we showed its predictive power of frailty in relatively healthy women. Interestingly, this group of women had poor scores on physical fitness [15th percentile on a normative scale; see Table 1 and (40)], and their score on physical activity was lower than that of the men and of the older women (Table 1). In addition, physical activity and fitness in this group were correlated.

It has been shown that exercise prevents frailty in mice and that frailty in institutionalized older people can be reversed by the treatment of exercise (41). It is possible that more physical activity and increased aerobic fitness in this group of young-old women may slow down the frailty process, which is expected to significantly increase at this early stage of frailty (6).

Importantly, sleep quality contributed even more than physical fitness to explaining the variability of frailty in the young old women, and it was also a significant contributor to explaining the variability of frailty in the old women. This finding supports the limited, although consistent, evidence on the relationship between sleep quality and frailty [e.g., (12, 13)]. Furthermore, our findings also support the literature reporting the modifying effect of gender on the association between sleep disturbances and frailty. It appears that the association between sleep disturbances and frailty only exists among female older adults (12). However, another review focusing on sleep durations reported no gender differences in the association between longer and shorter sleep durations and the increased risk of frailty (13). Further research is needed to explore the impact of sleep disturbances and quality (insomnia symptoms, excessive daytime sleepiness and sleep-wake patterns) on frailty in women as well as in men in early stages of frailty, possibly incorporating assessments and interventions for improving sleep quality in this group.

In addition to sleep, BMI was dominant in explaining the variability of frailty in older women. This finding supports the studies on overweight and frailty (24, 27), as opposed to the more acceptable approach of underweight as a sign of frailty (25). Oxidative stress was another component explaining frailty in older women that was also supported by previous literature (20). Oxidative stress, more than the other variables examined in this study, has been associated with aging. Furthermore, out of all factors influencing aging, such as inflammation, telomere shortening or degradation of proteins, the deteriorations related to oxidative stress rank first (20). It should be noted that oxidative stress has been linked to physical fitness (42). Given that the older women in this study performed very poorly on physical fitness (10th percentile), it is possible that their ability to counter the debilitating effect of oxidative stress is challenged, resulting in a higher level of H_2_O_2_. Therefore, increased aerobic fitness may affect the oxidative stress balance favoring the anti-oxidative cellular capacity.

Clearly, the directionality of the relationships between frailty and oxidative stress, between frailty and physical fitness and between frailty and sleep quality is still questionable. There are interrelationships between the potential correlates of frailty, reflecting the connections between different elements of the human organism (26). Additional research is required to confirm or refute the causative hypothesis on the effect of the variables examined in this study on frailty.

Correlates of frailty may include numerus variables, such as nutrition, genetics, living environment, etc., which were not included in this study. This may explain why the six variables assessed in the present study did not correlate with frailty in men, and collectively explained only 9.3% of the variability of frailty in men in our study. Perhaps there are other variables that can explain frailty in men, such as socioeconomic status (43).

A limitation of the present study is that it is not based on a representative sample of any older population. On the other hand, based on the demographic and clinical data, it is reasonable to assume that our participants resemble other populations of older adults who are better educated, healthier, and more active than other elderly people. In addition, as the study is based on volunteers, for unexplained reasons the men were older than the women. As the women also outnumbered the men, we solved this problem by dividing the women into two age groups: older women comparable in age to the men in the study, and younger women. In this manner we were able to compare the older women to men and to younger women.

Notably, research is needed to explore the mechanisms underlying frailty and to translate them to behavioral and clinical studies. In addition, rather than exploring the manifestations of frailty in people who are rated high on frailty indexes, it may be useful to start this exploration as early as possible—when it is still possible to attenuate or even reverse the process of deterioration. Furthermore, longitudinal studies on frailty correlates may shed more light on the contribution of these correlates to explaining the variability of frailty. For example, in the current study the level of physical fitness contributes to the variability of frailty in young women. It is suggested to further examine whether change in the level of physical fitness is related to change in frailty. It is possible that increased fitness will reduce frailty and vice versa.

## Data Availability Statement

The raw data supporting the conclusions of this article will be made available by the authors, without undue reservation.

## Ethics Statement

The studies involving human participants were reviewed and approved by Ethical approval for the study was obtained from the Ethics Committee of the Hillel Yaffe Medical Center (Hadera, Israel). The patients/participants provided their written informed consent to participate in this study.

## Author Contributions

YN formulated the study questions, designed the study, obtained Ethics Committee approval, supervised the data collection, assisted in the statistical analyses, interpreted the data, and wrote the manuscript. SB-Z suggested the method for assessing oxidative stress, supervised the urine samples collection, analyzed the urine samples, and assisted in the interpretation of the data. AZ formulated the statistical design, performed the statistical analysis, and interpreted the data. AD took a significant part in formulating the study questions, assisted in selecting the study measures, assisted in designing the study, coordinated the data collection, and assisted in the statistical analyses and in interpreting the data. All authors contributed to the article and approved the submitted version.

## Conflict of Interest

The authors declare that the research was conducted in the absence of any commercial or financial relationships that could be construed as a potential conflict of interest.
